# The role of small intestinal bacterial overgrowth and false positive diagnosis of lactose intolerance in southwest Hungary—A retrospective observational study

**DOI:** 10.1371/journal.pone.0230784

**Published:** 2020-05-08

**Authors:** Péter Varjú, Birgit Ystad, Noémi Gede, Péter Hegyi, Dániel Pécsi, József Czimmer

**Affiliations:** 1 Institute for Translational Medicine, Szentágothai Research Centre, Medical School, University of Pécs, Pécs, Hungary; 2 Division of Gastroenterology, First Department of Medicine, Medical School, University of Pécs, Pécs, Hungary; 3 Hungarian Academy of Sciences-University of Szeged, Momentum Gastroenterology Multidisciplinary Research Group, Szeged, Hungary; University of Witwatersrand/NHLS, SOUTH AFRICA

## Abstract

**Background:**

Lactose intolerance is a frequent gastrointestinal disease affecting 47% of the Eastern European population. Small intestinal bacterial overgrowth (SIBO) leads to carbohydrate malabsorption and therefore to false results during lactose breath and tolerance tests.

**Objectives:**

We aimed to assess the prevalence of lactose maldigestion and intolerance in Hungary and to investigate the role of combined diagnostic method and testing for SIBO in reducing false results.

**Methods:**

We retrospectively analyzed data from 264 adult symptomatic patients who underwent 50g lactose breath and tolerance tests in parallel over a one-year period at our center. A ≥20 ppm elevation of H_2_ or less than 1.1 mmol/l rise of blood glucose was diagnostic for lactose maldigestion. Patients with maldigestion who had symptoms during the test were defined as lactose intolerant. Patients with an early (≤90 min) significant (≥20 ppm) rise of H_2_ during lactose and/or lactulose breath tests were determined to have SIBO. Patients with slow/rapid oro-cecal transit and inappropriate preparation before the test were excluded.

**Results:**

49.6% of the 264 patients had lactose maldigestion, and 29.5% had lactose intolerance. The most frequent symptom was bloating (22.7%), while 34.8% of the study population and 60% of the symptomatic patients had SIBO. In 9.1% and 9.8% of the patients, the lactose breath and tolerance test alone gave false positive result compared with the combined method. SIBO was present in 75% of the false positives diagnosed with breath test only.

**Conclusions:**

The prevalence of lactose intolerance is lower in Hungary compared to the Eastern European value (29.5% vs 47%), so it is worth performing a population-based prospective analysis in this area. A combination of lactose breath and tolerance tests and the careful monitoring of results (with early H2 rise, lactulose breath test, etc.) can decrease the false cases caused by e.g. SIBO.

## Introduction

Lactose intolerance (LI) is a clinical syndrome characterized by abdominal symptoms after ingestion of lactose-containing products caused by lactose maldigestion (LM) [1–3]. The most common cause of primary LM is adult-type hypolactasia [4, 5]. Acquired organic disorders (e.g. small intestinal bacterial overgrowth (SIBO), celiac disease, inflammatory bowel disease (IBD), and infectious enteritis (e.g. giardiasis)), can lead to both downregulation of lactase expression and reduction of absorptive capacity and therefore to secondary lactose malabsorption [4, 5]. Approximately 47% of the Eastern European population is affected; however, LI is more common in Asia, Africa, and South America. It affects males and females equally [2, 6]. The prevalence of LM increases with age, however, the LI symptoms decrease in elderly [7, 8]. Because of insufficient lactase activity, lactose can reach the large intestine, where it is fermented by colonic bacteria, gases (H_2_, CO_2_, and CH_4_), short-chain fatty acids, and other products that are formed there. Excessive gas production causes luminal distension and leads to different gastrointestinal symptoms. The most common complaints are abdominal pain and discomfort, bloating, flatulence, and diarrhea as with SIBO [1, 3, 9–11]. The diagnostic methods available for LM or LI are based on the lactose breath test (LBT), lactose tolerance test (LTT), genetic test, and assessment of lactase activity in jejunal biopsy specimens, the LBT and LTT being the most popular methods [4]. However, in most studies and at most centers, only one of the last two methods (LBT or LTT) is used, resulting in higher rates of incorrect diagnosis caused by SIBO, for example, which can lead to carbohydrate malabsorption and therefore to false positive results during the LBT and LTT. Moreover, in some patients with methanogenic microbiota (e.g. Methanobrevibacter smithii), the bacteria convert hydrogen to methane, leading to false negative LBT results [2]. Restricting lactose intake or replacing the lactase enzyme can alleviate unpleasant lactose-induced symptoms [2–5].

SIBO is a condition in which the small intestine is excessively colonized by aerobic and anaerobic bacteria. Normally, there are fewer than 10^5^ bacteria per milliliter in the duodenal and jejunal part of the small intestine, with ileal counts reaching 10^8^ per milliliter [12]. The prevalence of SIBO is unclear, depending on the population and the diagnostic test used. It is more frequent among the elderly due to reduced gastric acid secretion and medications causing hypomotility [13]. Disorders disturbing mucosal defense mechanisms can predispose one to SIBO, intestinal motility disorders and chronic pancreatitis being the most common causes [14–16]. Other etiological factors are motility disorders (diabetes mellitus, irritable bowel syndrome [IBS], use of narcotics, intestinal pseudo-obstruction, etc.), anatomic disorders (adhesions, strictures, diverticulosis, etc.), immunological disorders (e.g. HIV), and metabolic and systemic diseases (e.g. cirrhosis) [12, 17–19]. SIBO causes mucosal damage and altered motility and therefore leads to complex malabsorption (of carbohydrate, fatty acids, proteins, and vitamins), diarrhea, bloating, flatulence, and abdominal discomfort [13, 20–23]. A diagnosis of this disease can be based on carbohydrate breath tests or on an assessment of bacterial concentration from the jejunal aspirate. Although jejunal aspirate culture is the gold standard method, it is not widely used due to its invasiveness, poor reproducibility, possible contamination, and patchy disease localization. Carbohydrate breath tests are simple, non-invasive, inexpensive, and therefore widely used [24–26]. The treatment comprises correction of the underlying cause, antibiotic therapy, and nutritional support (e.g. lactose-free diet, vitamin replacement, and correction of nutrient deficiencies). Rifaximine is effective in 80% of patients [27, 28]. Higher doses (1200 or 1600 mg/day) are more effective compared to standard ones (600 or 800 mg/day) [29]. The length of antibiotic therapy is not clearly defined. A single 7–10-day course can alleviate symptoms in most patients [30]. Repeated or continuous antibiotic therapy should be useful if symptoms recur [13]. The effectiveness of probiotics is inconclusive, and generally, they are not recommended in SIBO [18, 31].

In this single-center retrospective study, we aimed to assess the prevalence of LM and LI in southwest Hungary (Baranya County, except for the Mohács district, with a population of 317,000 people), to investigate the role of a combined diagnostic method (LBT and LTT) in improving diagnostic accuracy, and to show that parallel testing for SIBO could reduce false positive cases determined by LBT and/or LTT.

## Materials and methods

The key points of the STROBE (Strengthening the Reporting of Observational Studies in Epidemiology) guideline [32] were followed in planning and reporting this study (S1 File). We retrospectively analyzed data from adult symptomatic patients who underwent the LBT and LTT in parallel at our center (Division of Gastroenterology, First Department of Medicine, University of Pécs) between 15 February 2016 and 14 February 2017. The LBT and LTT were carried out with 50g lactose (equal to the content of 1 liter of milk), H_2_ levels were measured with Micro H_2_ instrument (Micro Medical Limited, P.O. Box 6. Rochester, Kent ME1 2AZ ENGLAND). Before lactose ingestion, baseline end-alveolar H_2_ and blood glucose levels were measured (0 min). Then patients drank the set amount of lactose dissolved in 250 ml water. After this process, end-alveolar H_2_ and blood glucose levels were measured every 30 minutes over a three-hour period (in the case of glucose over a two-hour period). Depending on the clinical situation and patients’ compliance, in clinically uncertain (but not in all) cases, a lactulose breath test with 10g lactulose was carried out to prove or reject the diagnosis of SIBO or slow oro-cecal transit [26]. A significant, ≥20 ppm elevation of H_2_ level during the LBT and/or less than 1.1 mmol/l rise of blood glucose during the LTT was diagnostic for LM. Patients with negative LBT and LTT are lactose digesters. Patients with LM who had symptoms during the test were defined as lactose intolerant. Patients with an early (≤90 min) significant (≥20 ppm) rise of H_2_ during the LBT and/or lactulose breath test were determined to have SIBO [26]. The diagnostic criteria of the different conditions are summarized in Table 1. For optimal preparation, patients stopped taking laxatives, antibiotics, and prokinetics, avoided high fiber-containing foods and fasted for 12 hours, avoided smoking and exercise for at least two hours before the test. Antiseptic mouthwash was not given routinely, only for those with high initial H_2_ value (>20 ppm). We excluded patients with inappropriate preparation for the test (baseline H_2_ level >20 ppm) and those with suspected rapid or slow oro-cecal transit (clinical symptoms of gastroparesis and a negative LBT with a positive LTT or no significant rise of H_2_ during a 180-min lactulose breath test compared to the baseline value). We collected data on the baseline characteristics of the analyzed population (mean age, gender differences, and their correlation with the outcome measures), the diagnostic tests (baseline and maximum H_2_ and glucose levels, time of glucose and H_2_ peak, and the presence of LM), the presence and type of symptoms occurring during the test (abdominal pain, cramps, discomfort, bloating, diarrhea, nausea/vomiting, borborygmi, and other gastrointestinal symptoms, such as increased bowel motility, flatulence, belching, a sensation of fullness in the stomach, a burning sensation in the stomach, an increased sensation for defecation or headache [3, 33]), and the presence of LI and SIBO. The data collection and research were approved by the director of the Clinical Center and the director of the First Department of Medicine of the University of Pécs (Institutional Review Board), and the study process was carried out in accordance current laws and regulations (Case Number: PTE/98494/2018). All patient data were fully anonymized after the specific parameters necessary for our research were collected. However, our analysis was made retrospectively; therefore, we have not included patients’ data who had refused scientific purpose data handling.

**Table 1:** The summary of the different diagnostic criteria used in our study.

**Lactose maldigester (LM)**	**LBT**: ≥20 ppm elevation of H_2_ compared to baseline level and/or **LTT**: <1.1 mmol/l rise of blood glucose level compared to baseline value
**Normal lactose digestion**	Negative LBT (<20 ppm elevation of H_2_ level) and Negative LTT (≥1.1 mmol/l elevation of blood glucose)
**Lactose intolerance (LI)**	Lactose maldigesters, who had symptoms during the test period
**Small intestinal bacterial overgrowth (SIBO)**	Significant (≥20 ppm) rise of H_2_ during lactose and/or lactulose breath test, within 90 minutes
**Slow oro-cecal transit (excluded)**	Clinical symptoms of gastroparesis and a negative LBT with a positive LTT or no significant rise of H_2_ during a 180-min lactulose breath test compared to the baseline value

LBT: lactose breath test; LTT: lactose tolerance test.

### Statistical analysis

Data were analyzed using SPSS 25.0 software. Means, standard deviation, minimum and maximum values, and relative frequency were calculated for descriptive statistics. The Pearson correlation, the Mann–Whitney test, and odds ratios (OR) with 95% confidence interval (CI) were used for other analyses. A p-value of less than 0.05 was accepted as statistically significant.

## Results

A total of 310 patients were assessed in the period noted above. Twenty-four of them were excluded because of inappropriate preparation and 22 (7.6% of the well-prepared patients) were ruled out because of slow oro-cecal transit, leaving 264 patients, 185 females (F: 70.1%), and 79 males (M: 29.9%), for statistical analysis. No patient had rapid transit in our study group. The mean age of the analyzed study group was 40.3 years (F: 40.6 years; M: 39.5 years).

**Fig 1:**
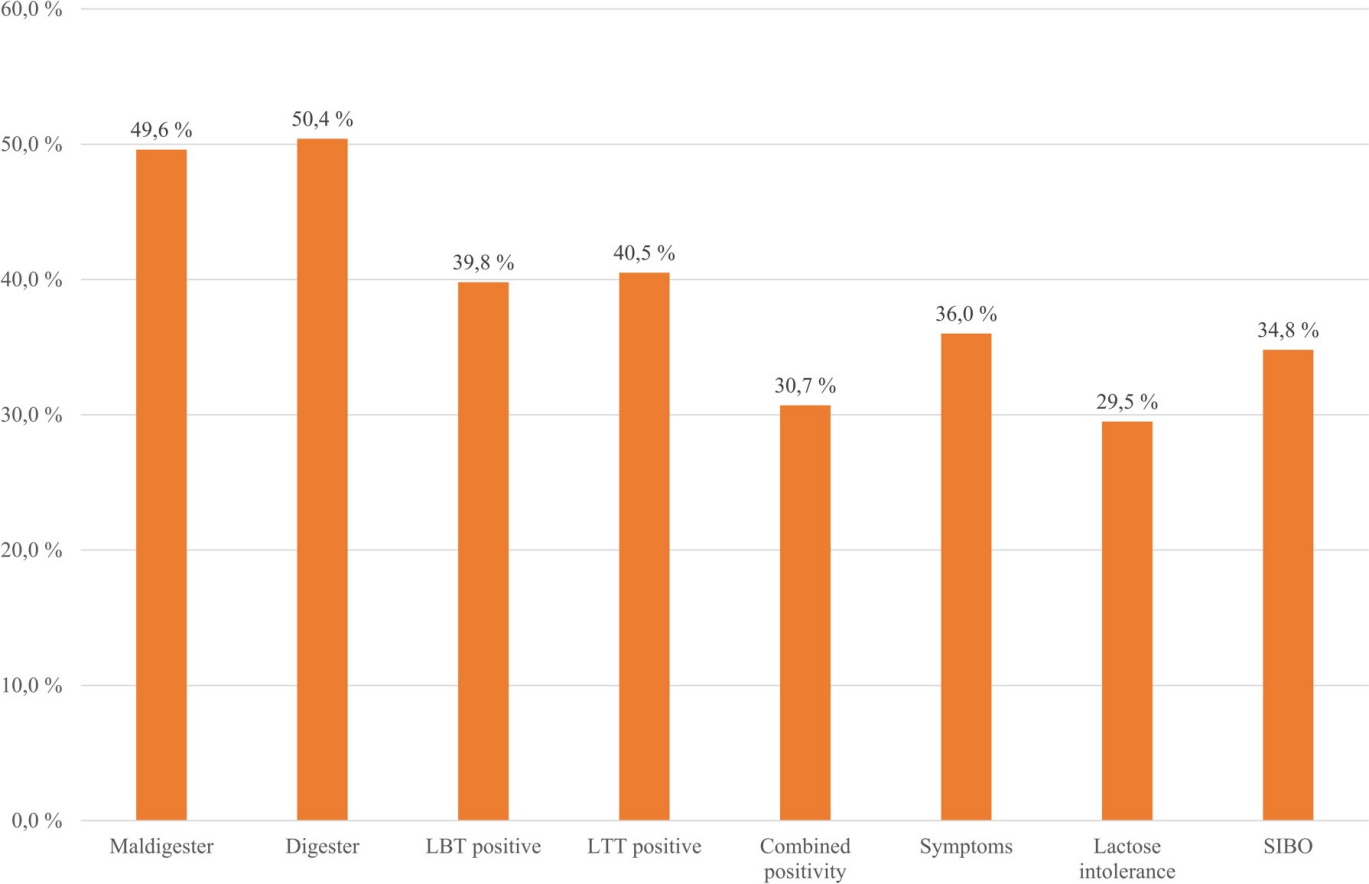
Summary of the basic results in the analyzed study population. A significant, ≥20 ppm elevation of H2 level during LBT and/or less than 1.1 mmol/l rise of blood glucose during LTT were diagnostic for lactose maldigestion. Patients with negative LBT and LTT are lactose digesters. Patients with LM who had symptoms during the test were defined as lactose intolerant. Patients with an early (≤90 min) significant (≥20 ppm) rise of H_2_ during LBT and/or lactulose breath test were defined to have SIBO. LBT: lactose breath test; LTT: lactose tolerance test; SIBO: small intestinal bacterial overgrowth.

Based on the LBT and/or LTT results, 49.6% (131/264) of the study population had LM (LBT and/or LTT positivity), as represented in Fig 1. Seventy-eight (78/131, 59.5%) of them had symptoms after lactose ingestion and were therefore defined as lactose intolerant (78/264, 29.5%, Fig 1). Combined positivity (LBT+LTT) was found in 30.7% (81/264) of the patients (see Fig 1). There was no significant difference between females and males in the prevalence of normal lactose digestion, LM, and LI (p > 0.05). There was no significant correlation between age and digester (p = 0.352), maldigester (p = 0.352), and LI (p = 0.098) status. The basic results of the analyzed population are summarized in Fig 1. The gender-related results are represented in S1 Fig.

### Lactose maldigestion and intolerance based on the LBT

**Fig 2:**
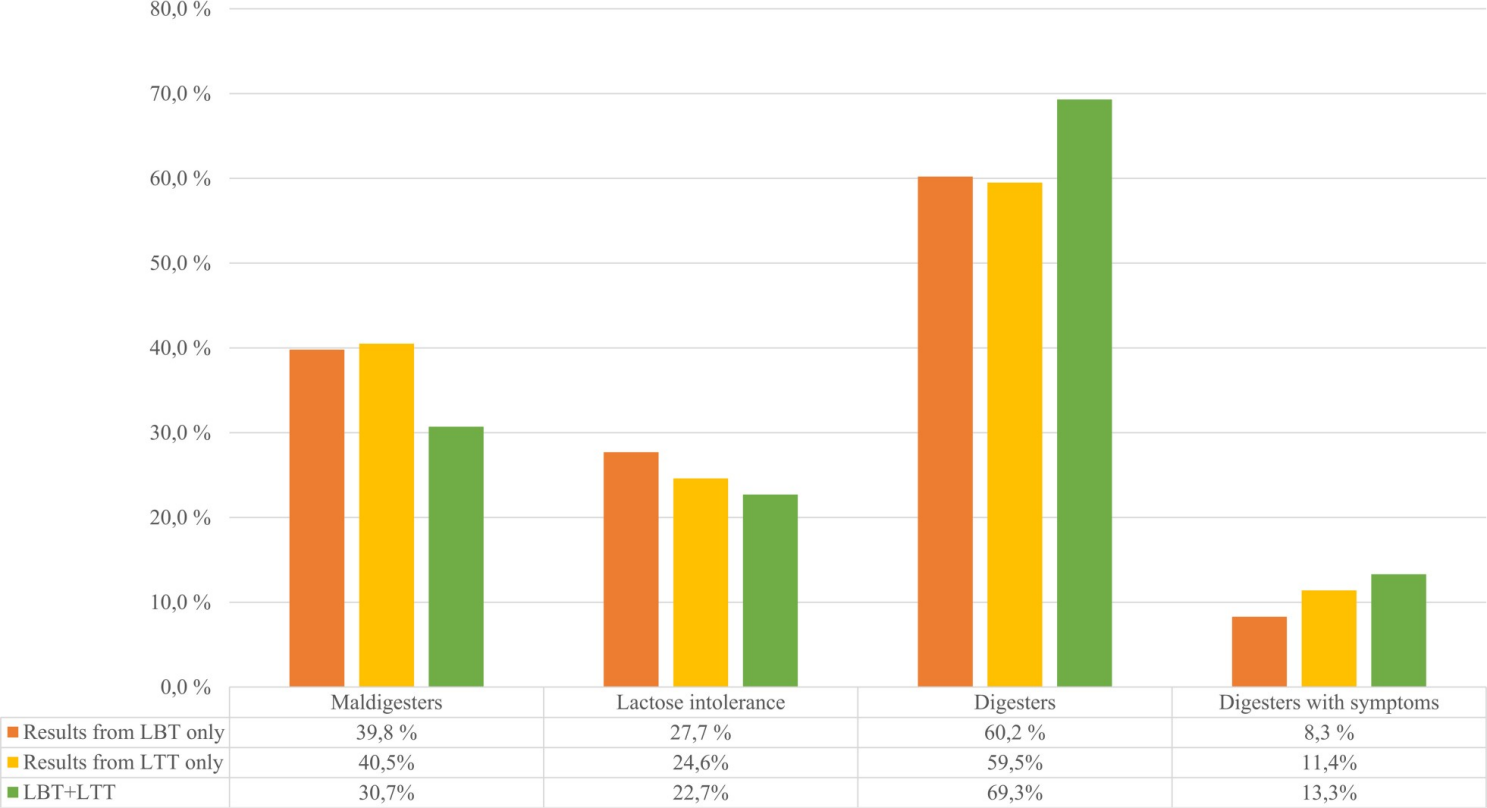
Summary of the results based separately on the LBT, LTT, and on the combination of them. LBT: lactose breath test; LTT: lactose tolerance test.

Based on the LBT only, 39.8% of the tested study population (105/264) were LM, and 73 of them (69.5%) had symptoms during the test; therefore, 27.7% (73/264) of the population was defined as lactose intolerant (see Fig 2). The majority (159/264, 60.2%) of the patients had a negative LBT, however; 13.8% (22/159) of them had symptoms after lactose ingestion, meaning that 8.3% (22/264) of the analyzed patients had symptoms without a positive test result, as represented in Fig 2. There was a weak negative correlation between age and baseline H_2_ (p = 0.009; r = -0.161). There was no significant connection between gender, age, and LBT positivity (gender: p > 0.05; age: p = 0.792). The results are summarized in Fig 2.

### Lactose maldigestion and intolerance based on the LTT

Based on an analysis of the LTT alone measured in parallel, 40.5% of the same study population (107/264) were maldigesters and 65 of them (60.7%) had symptoms during the test. Therefore, 24.6% (65/264) of the population was defined as lactose intolerant (see Fig 2). The majority (157/264, 59.5%) of the patients had a negative LTT; however, 19.1% (30/157) of them had symptoms after lactose ingestion, meaning that 11.4% (30/264) of the analyzed patients had symptoms without a positive test result (Fig 2). Men had a significantly higher baseline (p < 0.001) and maximum (p = 0.015) glucose level. There was a moderate positive correlation between age and glucose levels (baseline: p < 0.001; r = 0.338; maximum: p < 0.001; r = 0.222). There was no significant connection between gender, age, and LTT positivity (gender: p > 0.05; age: p = 0.378). The results are summarized in Fig 2.

### Combined LHBT and LTT positivity

Combined positivity (LBT+LTT) was found in 30.7% (81/264) of the patients, 74% of them (60/81) had symptoms. Therefore, 22.7% (60/264) of the study population was lactose intolerant based on the combined results (see Fig 2). In the majority (183/264, 69.3%) of the population one or both tests were negative; however, 19.1% (35/183) of them had symptoms meaning that 13.3% (35/264) of the analyzed patients had symptoms without combined test positivity (Fig 2). The results are summarized in Fig 2.

### Clinical symptoms

**Fig 3:**
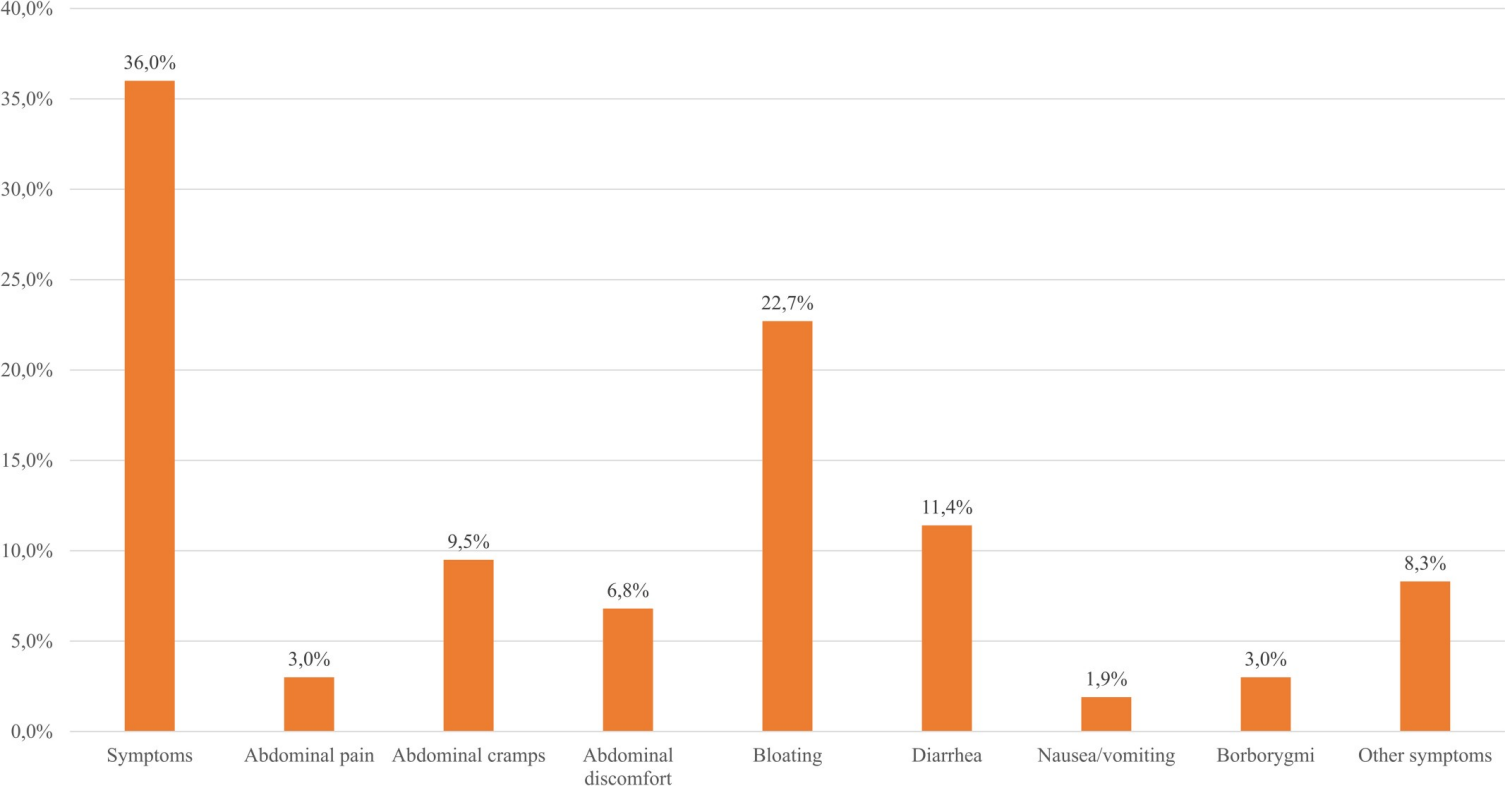
The frequency and distribution of different symptoms in the entire study population. Other symptoms comprise increased bowel motility, flatulence, belching, sensation of fullness in the stomach, headache, burning sensation in the stomach, or increased sensation for defecation.

**Fig 4:**
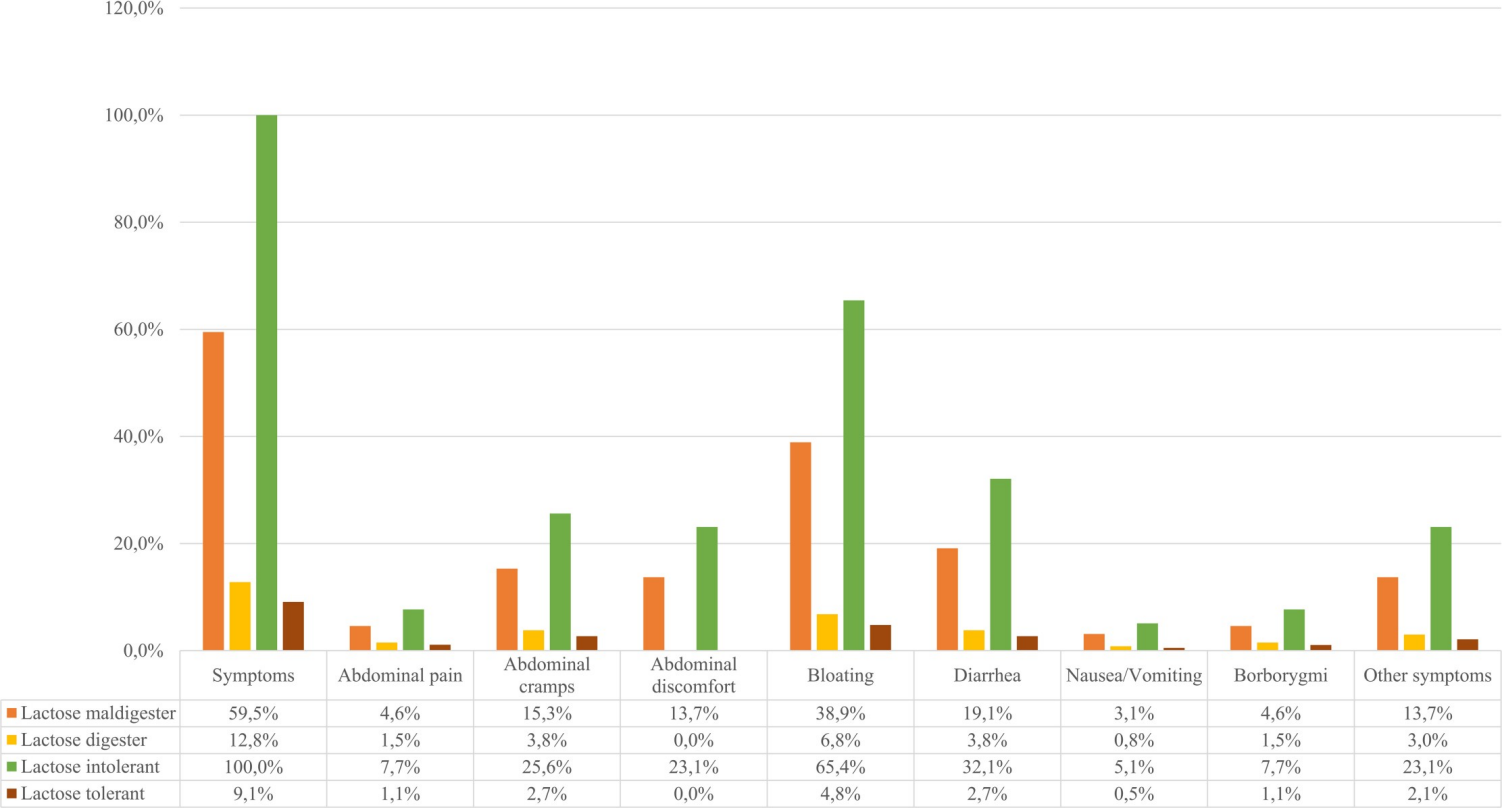
The frequency and distribution of different symptoms among lactose digesters/maldigesters, and among lactose tolerant/intolerant patients. A significant, ≥20 ppm elevation of H2 level during LBT and/or less than 1.1 mmol/l rise of blood glucose during LTT were diagnostic for lactose maldigestion. Patients with negative LBT and LTT are lactose digesters. Patients with LM who had symptoms during the test were defined as lactose intolerant. Other symptoms comprise increased bowel motility, flatulence, belching, sensation of fullness in the stomach, headache, burning sensation in the stomach, or increased sensation for defecation.

Thirty-six percent (95/264) of the patients had symptoms after lactose ingestion (see Fig 1), bloating being the most frequent (60/264; 22.7%), as seen in Fig 3. There was no statistically significant difference between females and males in the presence of symptoms (p > 0.05). Those who had nausea/vomiting were significantly older (p = 0.014). Otherwise, there was no statistically significant correlation between age and symptoms (p = 0.204). 12.8% (17/133) of the lactose digester patients (the LBT and LTT are negative) and 59.5% (78/131) of the maldigester patients (at least one of the tests is positive) had clinical symptoms (see Fig 4). Based on the latest meta-analysis conducted by our workgroup [34], we hypothesize that IBS may be a contributing factor in LI among lactose maldigesters. Figs 3 and 4 show the frequency of the different symptoms in the study population, and among lactose maldigesters/digesters and lactose intolerant/tolerant patients. Female/male data regarding symptoms are represented in S2 Fig.

### The role of SIBO

Approximately one-third (92/264; 34.8%) of the study population (see Fig 1) and 60% (57/95) of the symptomatic patients had SIBO based on the definition (see Table 1). There was no significant difference in the presence of SIBO between females and males (F: 68/185, 36.8%; M: 24/79, 30.4%, p > 0.05); furthermore, there was no significant correlation between age and SIBO (p = 0.848). SIBO patients had significantly higher maximum H_2_ levels (p < 0.001), and they reached the H_2_ peak later (p < 0.001). Moreover, they had lower maximum glucose levels (p < 0.001), and LTT positivity was significantly more frequent in this patient group (OR = 5.833; 95% CI: 3.356–10.138). Symptoms were more common in SIBO patients compared to non-SIBO patients (OR = 5.743; 95% CI: 3.300–9.994), especially abdominal discomfort (OR = 3.201; 95% CI: 1.196–8.565), bloating (OR = 4.798; 95% CI: 2.606–8.833), diarrhea (OR = 6.443; 95% CI: 2.737–15.168), and other symptoms (OR = 5.825; 95% CI: 2.193–15.469).

**Fig 5:**
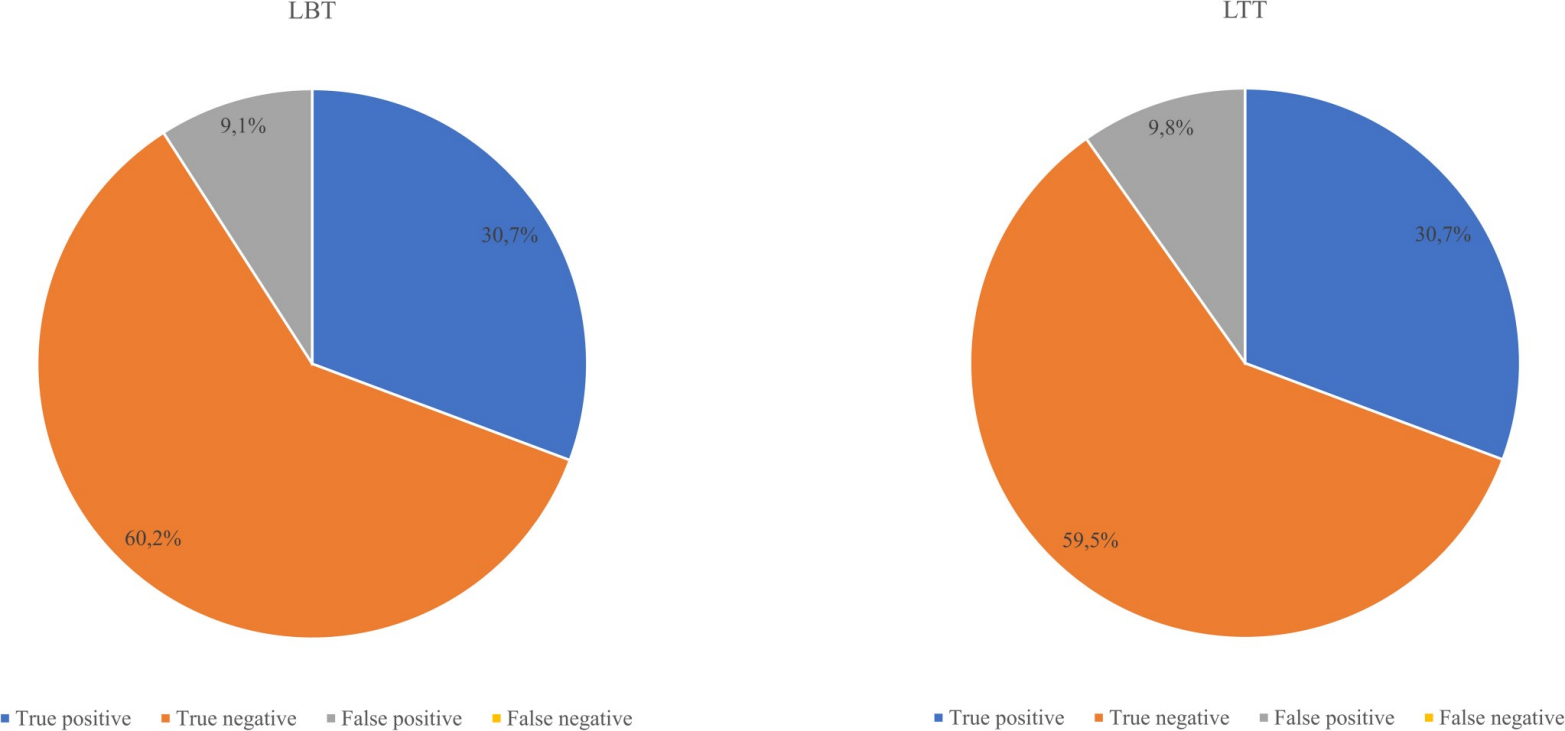
The diagnostic accuracy of the LBT and LTT verified by the combined results of the tests. LBT: lactose breath test; LTT: lactose tolerance test.

In 90.9% (240/264) of the patients the LBT gave correct diagnosis (30.7% true positive: 81/264, 60.2% true negative: 159/264) of LM (or the lack of it) using combined LBT and LTT as reference. False positive results were found in 9.1% (24/264) of the cases; however, there are no false negatives in this setting (see Fig 5). LBT in this setting has 100% sensitivity, 86.9% specificity, 77.1% positive predictive value, and 100% negative predictive value. SIBO was found in 76.5% (62/81) of the true positive and in 75% (18/24) of the false positive patients.

In 90.2% (238/264) of the patients the LTT gave correct diagnosis (30.7% true positive: 81/264, 59.5% true negative: 157/264) of LM (or the lack of it) using combined LBT and LTT as reference. False positive results were found in 9.8% (26/264) of the cases; however, there are no false negatives in this setting (see Fig 5). Therefore, LTT has 100% sensitivity, 85.8% specificity, 75.7% positive predictive value, and 100% negative predictive value. SIBO was found in 76.5% (62/81) of the true positive, but surprisingly in 0% (0/26) of the false positive patients.

Based on these findings the combination of the LBT and LTT and the careful monitoring of results (with e.g. early H_2_ rise, parallel performed lactulose breath test) can decrease false results caused by e.g. SIBO.

## Discussion

In this retrospective, single-center study, we analyzed the epidemiological characteristics of LI in southwest Hungary and assessed the role of combined diagnostic method and small intestinal bacterial overgrowth in the accuracy of the diagnosis.

LI is a relatively common problem in the white population, affecting approximately 47% of Eastern European adults [2, 6]. There are widely-used, inexpensive, non-invasive, diagnostic methods based on measurement of end-alveolar H_2_ concentration (LBT) or blood glucose (LTT) [2–5]. The sensitivity and specificity of these tests are relatively high, but they depend on the ingested lactose dose (25g LBT: 82% and 95%; 25g LTT: 78% and 93%; 50g LBT: 92% and 83%; 50g LTT: 94% and 90%) [35, 36]. Other circumstances, such as SIBO, antibiotic usage, lung diseases, inappropriate preparation, and abnormal gastric emptying can influence their diagnostic accuracy. A combination of these tests and careful evaluation of the results can reduce the false positive or negative cases; however, in most studies, they are used separately [37].

The gold standard diagnostic method is the testing of lactase activity in duodenal and jejunal biopsy samples taken from the mucosa. However, due to the invasiveness, high costs, and patchy enzyme expression, it is less frequently performed compared to the tests noted above. Moreover, it should be considered that similar lactase activity in two patients might result in different LBT results due to the different activity and composition of the intestinal microbiota. There are several genes associated with lactase non-persistence (C/T_13910 with CC genotype; G/A_22018 with GG genotype), but the availability of genetic testing is variable, and its costs are relatively high. Moreover, the lactase non-persistence allele is not always associated with LM [2, 3, 5, 34]. A Hungarian study, published by Nagy et al., determined the applicability of the LBT in comparison with genetic screening (C/T_13910). They found that 37% of the analyzed population had lactase non-persistence, which correlated well with positive LBT results is symptomatic children [38]. We found similar LBT positivity among symptomatic adults. Another retrospective study from Hungary, conducted by Buzás et al., also underlined that both genetic and breath tests are sufficiently accurate [39].

In this study, we presented epidemiological data on the prevalence of LM and LI in southwest Hungary, we analyzed the frequency of the most common symptoms, we demonstrated that combined analysis of LBT and LTT can improve diagnostic accuracy and the parallel testing for SIBO could reduce false cases caused for example by SIBO. It should also be mentioned that the study population had a very large female representation (185 vs 79); however, there were no statistically significant gender-related differences regarding LM, LI, LBT/LTT positivity, symptoms frequency, and prevalence of SIBO, which underlines the literature data in case of LI [2]. Moreover, despite the literature data [7, 8, 13], we did not find any age-related correlations in the outcomes mentioned above.

The limitations of our results should be considered for a correct interpretation, thus possibly influencing outcomes. Firstly, our results are based on a single-center retrospective medical database analysis. Secondly, we only analyzed the results in a one-year period; therefore, the number of enrolled patients is relatively low. Thirdly, the amount of ingested lactose can influence the prevalence of LM and LI, and the frequency of symptoms. We used a relatively high dose of lactose and we did not perform blinded testing with placebo. Based on the retrospective character, follow-up after antibiotic treatment or low-lactose diet could not be performed to confirm the diagnosis of SIBO and LI based on symptom relief. Moreover, lactulose breath test was performed only in clinically uncertain cases, not on all patients. Therefore, the true diagnosis and prevalence of LI and SIBO could not be assessed correctly. Only patients with high initial end-alveolar H_2_ concentration got antiseptic mouthwash. Another significant limitation is that our study group comprises symptomatic patients referred to our clinic, thus potentially leading to sampling bias. It also should be considered that we did not measure methane levels in the end-alveolar gas samples to determine false negative LBT caused by methane producing bacteria. Based on the recent results [40] false negative LBT (5–15%) are mainly caused by methane production. Finally, the symptoms of the patients are subjective, thus possibly prompting inaccurate conclusions. Interpretation of patient-reported symptoms will differ between clinicians; therefore, standardized symptom definitions should have been used to minimize errors. According to the Oxford Centre for Evidence-Based Medicine 2011, the evidence level of our findings is level 3 [41].

## Conclusions

Based on our results, we can conclude that the prevalence of LI is lower in Hungary compared to the Eastern European value (29.5% vs 47%) and that it is worth performing a population-based prospective analysis in this area. During the provocation tests, 59.5% of lactose maldigesters had IBS-like symptoms (lactose intolerance), but the role of IBS in the background is unknown. SIBO was relatively common among symptomatic patients (60%), and this may influence the diagnostic accuracy of lactose maldigestion, based on the LBT and LTT as the only diagnostic test. Therefore, a combination of the LBT and LTT and careful monitoring of results may decrease the false cases caused by e.g. SIBO.

## Supporting information

S1 File STROBE checklist. STROBE: Strengthening the Reporting of Observational Studies in Epidemiology [32].
(DOCX)

S1 Fig The summary of the basic results among females/males. A significant, ≥20 ppm elevation of H2 level during LBT and/or less than 1.1 mmol/l rise of blood glucose during the LTT were diagnostic for lactose maldigestion. Patients with negative LBT and LTT are lactose digesters. Patients with LM who had symptoms during the test were defined as lactose intolerant. Patients with an early (≤90 min) significant (≥20 ppm) rise of H_2_ during LBT and/or lactulose breath test were defined to have SIBO. LBT: lactose breath test; LTT: lactose tolerance test; SIBO: small intestinal bacterial overgrowth.
(TIF)

S2 Fig The frequency and distribution of different symptoms among females/males. Other symptoms comprise increased bowel motility, flatulence, belching, sensation of fullness in the stomach, headache, burning sensation in the stomach, or increased sensation for defecation.
(TIF)

## Abbreviations

CI: confidence interval
HIV: human immunodeficiency virus
IBD: inflammatory bowel disease
IBS: irritable bowel syndrome
LBT: lactose breath test
LI: lactose intolerance
LM: lactose maldigestion
LTT: lactose tolerance test
SIBO: small intestinal bacterial overgrowth
OR: odds ratio
