# Supplementary figures and images for: The role of small intestinal bacterial overgrowth and false positive diagnosis of lactose intolerance in southwest Hungary—A retrospective observational study
Source: PLoS One. 2020 May 8;15(5):e0230784. doi: 10.1371/journal.pone.0230784 (PMC7209350; doi:10.1371/journal.pone.0230784)

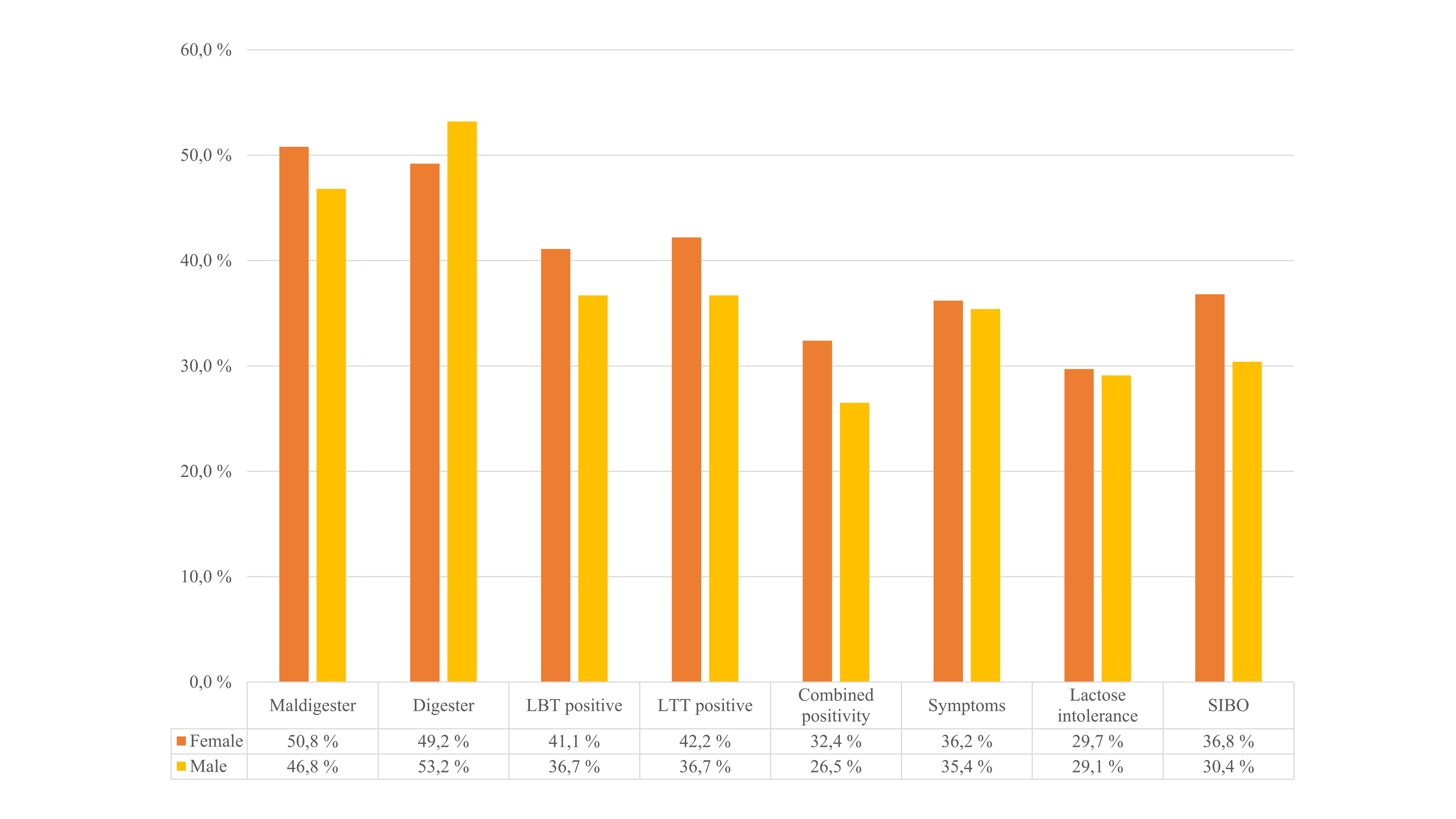

Supplement: S1 Fig — A significant, ≥20 ppm elevation of H2 level during LBT and/or less than 1.1 mmol/l rise of blood glucose during the LTT were diagnostic for lactose maldigestion. Patients with negative LBT and LTT are lactose digesters. Patients with LM who had symptoms during the test were defined as lactose intolerant. Patients with an early (≤90 min) significant (≥20 ppm) rise of H2 during LBT and/or lactulose breath test were defined to have SIBO. LBT: lactose breath test; LTT: lactose tolerance test; SIBO: small intestinal bacterial overgrowth. (TIF) [file pone.0230784.s002.tif]

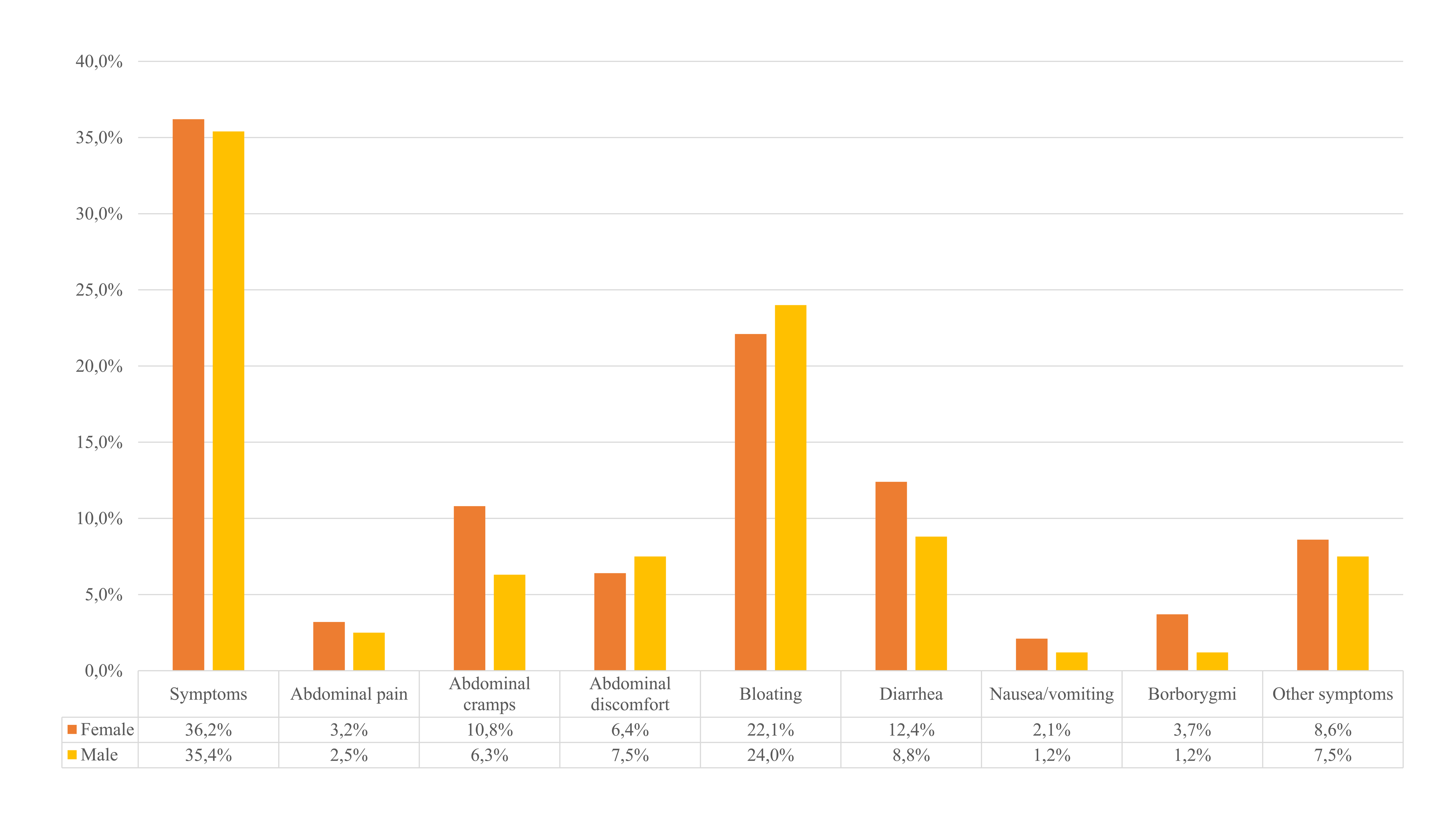

Supplement: S2 Fig — Other symptoms comprise increased bowel motility, flatulence, belching, sensation of fullness in the stomach, headache, burning sensation in the stomach, or increased sensation for defecation. (TIF) [file pone.0230784.s003.tif]
